# Draft Genome Sequences of Pseudomonas syringae pv. tomato Strains J4 and J6, Isolated in Florida

**DOI:** 10.1128/MRA.00127-21

**Published:** 2021-04-15

**Authors:** Naweena Thapa, Sujan Timilsina, Gerald V. Minsavage, Juliana Pereira-Martin, Gary E. Vallad, Erica M. Goss, Pamela Roberts, Zhonglin Mou, Shouan Zhang, Jeffrey B. Jones

**Affiliations:** a Plant Pathology Department, University of Florida, Gainesville, Florida, USA; b Gulf Coast Research and Education Center, University of Florida, Balm, Florida, USA; c Emerging Pathogens Institute, University of Florida, Gainesville, Florida, USA; d Southwest Florida Research and Education Center, Immokalee, Florida, USA; e Microbiology and Cell Sciences, University of Florida, Gainesville, Florida, USA; f Tropical Research and Education Center, University of Florida, Homestead, Florida, USA; Loyola University Chicago

## Abstract

Pseudomonas syringae pv. tomato causes bacterial speck in tomato. We report the genome sequences of two P. syringae pv. tomato strains, J4 and J6, that are genetically closely related, with >99.9 average nucleotide identity (ANI), but vary in the presence of coronatine-associated genes.

## Abstract (precis)

Pseudomonas syringae pv. Tomato causes bacterial speck in tomato. We report the genome sequences of two P. syringae pv. Tomato strains, J4 and J6, that are genetically closely related, with >99.9 average nucleotide identity (ANI), but vary in the presence of coronatine-associated genes.

## ANNOUNCEMENT

*Pseudomonas* is a Gram-negative genus of bacteria that belongs to the *Gammaproteobacteria* in the *Pseudomonadaceae* family. This genus contains more than 220 validly published species that inhabit diverse environmental niches and are associated with human and plant diseases (1–3). Pseudomonas syringae pv. tomato causes bacterial speck disease in tomato and requires a type III secretion system to infect and colonize the host (4). Additionally, P. syringae pv. tomato produces the phytotoxin coronatine, which functions as a defense suppressor (5). Coronatine is reported to mimic methyl jasmonate and promote P. syringae pv. tomato virulence and is important for symptom development resulting in chlorotic lesions in host plants (6–8) and inducing stomatal opening to facilitate entry into stomates (9–11).

**FIG 1:**
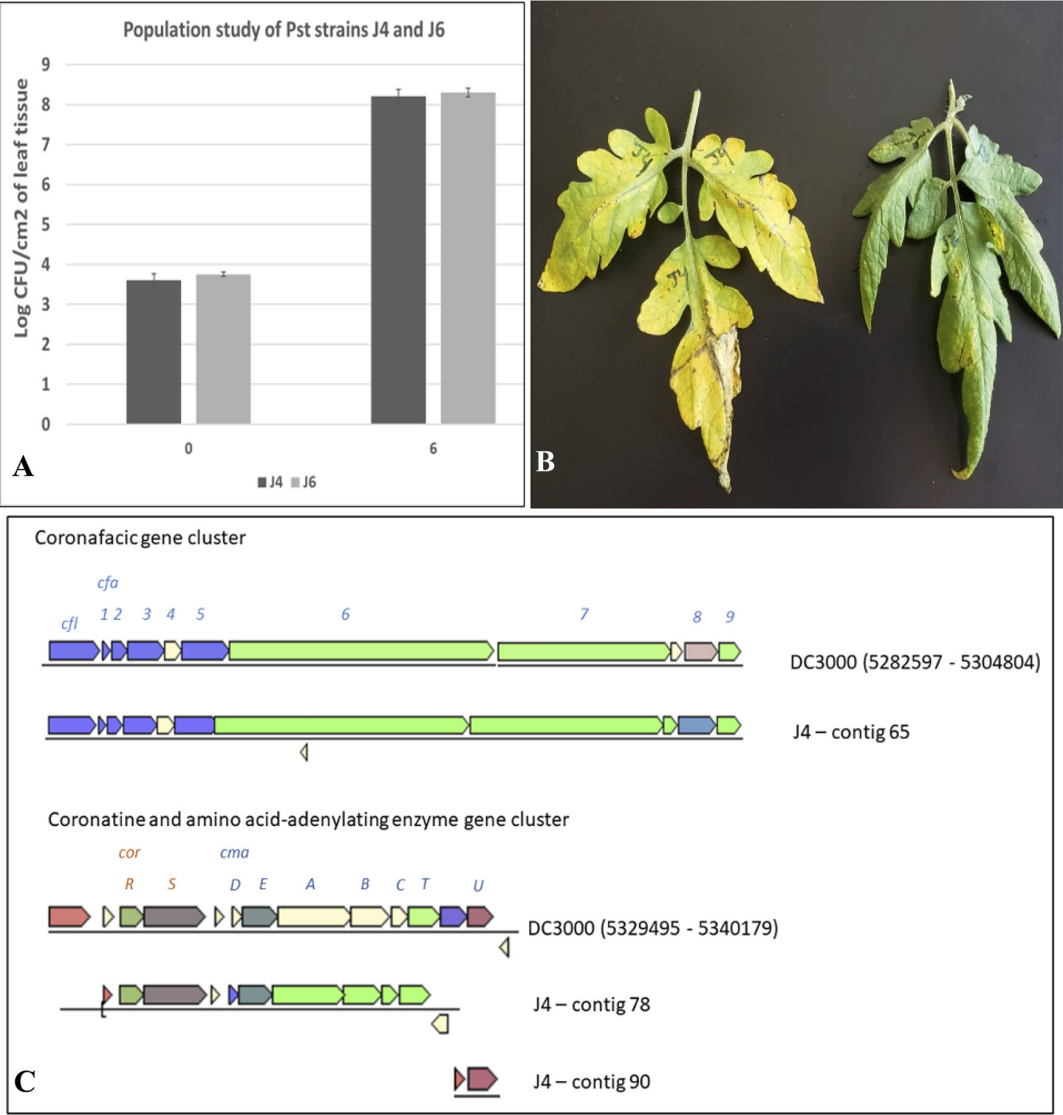
(A) Population study comparison of Pseudomonas syringae pv. tomato strains J4 and J6 in tomato. Bacteria multiplied to similar populations 6 days postinoculation. (B) Disease symptom development after inoculation with strains J4 (left) and J6 (right). High yellowing and chlorosis were observed in leaflets inoculated with strain J4 6 days postinoculation. For both experiments, the Bonny Best tomato cultivar was used. Leaves were infiltrated with bacterial suspension at ∼10^5^ CFU/ml for the population study, while dip inoculation was done with bacterial suspension at ∼10^8^ CFU/ml for monitoring the symptom development. (C) Coronafacic and coronatine gene clusters found in DC3000 and J4 strains of Pseudomonas syringae pv. tomato. The genomic cluster is missing in strain J6.

In April 2010, two P. syringae pv. tomato strains, J4 and J6, were isolated from tomato fields in Florida using a standard isolation procedure (12). Pathogenicity was confirmed in tomato, and the *in planta* bacterial populations were quantified by infiltrating strains at ∼10^5^ CFU/ml into tomato leaves (11). Populations of both J4 and J6 were similar 6 days postinfiltration (Fig. 1A). Additionally, tomato plants were dip inoculated using suspensions of both strains at ∼10^8^ CFU/ml. Interestingly, chlorosis was observed in J4- but not J6-inoculated plants (Fig. 1B). To investigate differences, J4 and J6 were subjected to whole-genome sequencing.

Genomic DNA was extracted from cultures grown in nutrient broth for 24 h using a Wizard genomic DNA purification kit (Promega, Chicago, IL). The genomic library was prepared using a Nextera DNA library preparation kit (Illumina, San Diego, CA). Sequencing was performed at the Interdisciplinary Center for Biotechnology Research, University of Florida, using Illumina MiSeq technology, generating 251-bp paired-end reads for each sample. Raw sequences were assembled using a previously described pipeline (13, 14). Briefly, raw reads were trimmed and paired with Trim Galore (15) and then assembled into contigs with Spades v.3.10.1 (16). Contigs smaller than 500 bp and with k-mer coverage less than 2.0 were removed. Validated reads were mapped to filtered contigs using Bowtie 2 v.2.3.3 (17). SAMtools was used for file conversion, and Pilon v.1.22 was used to polish the draft assembly to generate an improved FASTA file (18, 19). Genome assemblies were annotated using the Prokaryotic Genome Annotation Pipeline v.4.13 from the National Center for Biotechnology Information (20).

Genome statistics for J4 and J6 are provided in Table 1. Average nucleotide identity (ANI) based on BLAST, computed using JSpecies (21), showed >99.97% sequence identity between J4 and J6. Meanwhile, ANIs with a representative P. syringae pv. tomato strain, DC3000 (GenBank accession number GCF_000007815.1) were 98.64% and 98.63% for J4 and J6, respectively. Genome annotations of J4 and J6 were compared with that of DC3000 to identify the coronatine coding cluster using the JGI platform (https://img.jgi.doe.gov). J6 lacks the genomic region that encompasses the coronatine and coronafacic genes involved in coronatine production. However, J4 and DC3000 share similar genomic clusters necessary for coronatine production (Fig. 1C). Comparison of the genomic region encompassing the coronatine-associated genes (∼33 kb) indicated more than 97% sequence identity between J4 and DC3000. Previous studies showed that following inoculation with DC3000, stomates closed and later reopened, while with J4, stomates remained open (10, 11). Interestingly, with J6, stomates stayed open but later closed, supporting the possible importance of coronatine for keeping stomates open.

**TABLE 1:** Sequencing and genome statistics for Pseudomonas syringae pv. tomato strains J4 and J6

Characteristic	Data for strain:	
	J4	J6
Total no. of reads	603,606	858,404
Genome length (bp)	6,334,619	6,264,625
Genome coverage (×)	23.9	34.4
No. of contigs	151	111
Total no. of genes	5,678	5,591
*N*_50_ (bp)	112,230	138,773
GC content (%)	58.6	58.6

### Data availability.

The whole-genome sequence assemblies for J4 and J6 are deposited in GenBank under accession numbers JADODR000000000 and JADCNI000000000, respectively. The raw data are available under SRA numbers SRR12817639 for J4 and SRR12817638 for J6.

## References

[1] Parte AC. 2018. LPSN—list of prokaryotic names with standing in nomenclature (Bacterio.net), 20 years on. Int J Syst Evol Microbiol 68:1825–1829. doi:10.1099/ijsem.0.002786. 29724269

[2] Lalucat J, Mulet M, Gomila M, García-Valdés E. 2020. Genomics in bacterial taxonomy: impact on the genus *Pseudomonas*. Genes (Basel) 11:139. doi:10.3390/genes11020139. PMC7074058 32013079

[3] Mena KD, Gerba CP. 2009. Risk assessment of *Pseudomonas aeruginosa* in water. Rev Environ Contam Toxicol 201:71–115. doi:10.1007/978-1-4419-0032-6_3. 19484589

[4] Preston GM. 2000. *Pseudomonas syringae* pv. tomato: the right pathogen, of the right plant, at the right time. Mol Plant Pathol 1:263–275. doi:10.1046/j.1364-3703.2000.00036.x. 20572973

[5] Geng X, Cheng J, Gangadharan A, Mackey D. 2012. The coronatine toxin of *Pseudomonas syringae* is a multifunctional suppressor of *Arabidopsis* defense. Plant Cell 24:4763–4774. doi:10.1105/tpc.112.105312. 23204405 PMC3531865

[6] Bender CL, Alarcón-Chaidez F, Gross DC. 1999. *Pseudomonas syringae* phytotoxins: mode of action, regulation, and biosynthesis by peptide and polyketide synthetases. Microbiol Mol Biol Rev 63:266–292. doi:10.1128/MMBR.63.2.266-292.1999. 10357851 PMC98966

[7] Kenyon J, Turner JG. 1990. Physiological changes in *Nicotiana tabacum* leaves during development of chlorosis caused by coronatine. Physiol Mol Plant Pathol 37:463–477. doi:10.1016/0885-5765(90)90037-X.

[8] Chakravarthy S, Worley JN, Montes-Rodriguez A, Collmer A. 2018. *Pseudomonas syringae* pv. tomato DC3000 polymutants deploying coronatine and two type III effectors produce quantifiable chlorotic spots from individual bacterial colonies in *Nicotiana benthamiana* leaves. Mol Plant Pathol 19:935–947. doi:10.1111/mpp.12579. 28677296 PMC6637995

[9] Melotto M, Underwood W, Koczan J, Nomura K, He SY. 2006. Plant stomata function in innate immunity against bacterial invasion. Cell 126:969–980. doi:10.1016/j.cell.2006.06.054. 16959575

[10] Zeng W, Melotto M, He SY. 2010. Plant stomata: a checkpoint of host immunity and pathogen virulence. Curr Opin Biotechnol 21:599–603. doi:10.1016/j.copbio.2010.05.006. 20573499 PMC2946497

[11] Pereira JA, Yu F, Zhang Y, Jones JB, Mou Z. 2018. The *Arabidopsis* elongator subunit ELP3 and ELP4 confer resistance to bacterial speck in tomato. Front Plant Sci 9:1066. doi:10.3389/fpls.2018.01066. 30087688 PMC6066517

[12] Timilsina S, Minsavage GV, Preston J, Newberry EA, Paret ML, Goss EM, Jones JB, Vallad GE. 2018. *Pseudomonas floridensis* sp. nov., a bacterial pathogen isolated from tomato. Int J Syst Evol Microbiol 68:64–70. doi:10.1099/ijsem.0.002445. 29148362

[13] Fulton JC, Klein JM, Bec S, Fayette J, Garrett KA, Jones JB, Timilsina S, Harmon CL. 2020. Draft genome sequences of plant-pathogenic *Klebsiella variicola* strains isolated from plantain in Haiti. Microbiol Resour Announc 9:e00336-20. doi:10.1128/MRA.00336-20. 32675179 PMC7365790

[14] Timilsina S, Pereira-Martin JA, Minsavage GV, Iruegas-Bocardo F, Abrahamian P, Potnis N, Kolaczkowski B, Vallad GE, Goss EM, Jones JB. 2019. Multiple recombination events drive the current genetic structure of *Xanthomonas perforans* in Florida. Front Microbiol 10:448. doi:10.3389/fmicb.2019.00448. 30930868 PMC6425879

[15] Martin M. 2011. Cutadapt removes adapter sequences from high-throughput sequencing reads. EMBnet J 17:10. doi:10.14806/ej.17.1.200.

[16] Prjibelski A, Antipov D, Meleshko D, Lapidus A, Korobeynikov A. 2020. Using SPAdes de novo assembler. Curr Protoc Bioinforma 70:e102. doi:10.1002/cpbi.102. 32559359

[17] Langmead B, Salzberg SL. 2012. Fast gapped-read alignment with Bowtie 2. Nat Methods 9:357–359. doi:10.1038/nmeth.1923. 22388286 PMC3322381

[18] Li H, Handsaker B, Wysoker A, Fennell T, Ruan J, Homer N, Marth G, Abecasis G, Durbin R, 1000 Genome Project Data Processing Subgroup. 2009. The Sequence Alignment/Map format and SAMtools. Bioinformatics 25:2078–2079. doi:10.1093/bioinformatics/btp352. 19505943 PMC2723002

[19] Walker BJ, Abeel T, Shea T, Priest M, Abouelliel A, Sakthikumar S, Cuomo CA, Zeng Q, Wortman J, Young SK, Earl AM. 2014. Pilon: an integrated tool for comprehensive microbial variant detection and genome assembly improvement. PLoS One 9:e112963. doi:10.1371/journal.pone.0112963. 25409509 PMC4237348

[20] Tatusova T, DiCuccio M, Badretdin A, Chetvernin V, Nawrocki EP, Zaslavsky L, Lomsadze A, Pruitt KD, Borodovsky M, Ostell J. 2016. NCBI Prokaryotic Genome Annotation Pipeline. Nucleic Acids Res 44:6614–6624. doi:10.1093/nar/gkw569. 27342282 PMC5001611

[21] Richter M, Rosselló-Móra R. 2009. Shifting the genomic gold standard for the prokaryotic species definition. Proc Natl Acad Sci U S A 106:19126–19131. doi:10.1073/pnas.0906412106. 19855009 PMC2776425

